# Resistance and virulence gene analysis and molecular typing of *Escherichia coli* from duck farms in Zhanjiang, China

**DOI:** 10.3389/fcimb.2023.1202013

**Published:** 2023-06-15

**Authors:** Shuaishuai Luo, Cuiyi Liao, Jinju Peng, Songruo Tao, Tengyue Zhang, Yue Dai, Yuexia Ding, Yi Ma

**Affiliations:** ^1^ Department of Veterinary Medicine, College of Coastal Agricultural Sciences, Guangdong Ocean University, Zhanjiang, China; ^2^ College of Traditional Chinese Medicine, Zhanjiang University of Science and Technology, Zhanjiang, China; ^3^ Maoming Branch, Guangdong Laboratory for Lingnan Modern Agriculture, Maoming, China

**Keywords:** *Escherichia coli*, drug resistance gene, virulence gene, molecular typing, duck farm

## Abstract

**Introduction:**

The widespread use of antibiotics in animal agriculture has increased the resistance of *Escherichia coli*, and pathogenic *E. coli* often harbor complex virulence factors. Antimicrobial resistance in pathogenic bacteria can cause public health problems. Correlation analyses of the resistance, virulence, and serotype data from the pathogenic bacteria found on farms and in the surrounding environment can thus provide extremely valuable data to help improve public health management.

**Methods:**

In this investigation, we have assessed the drug resistance and virulence genes as well as the molecular typing characteristics of 30 *E. coli* strains isolated from duck farms in the Zhanjiang area of China. Polymerase chain reaction was used to detect the drug resistance and virulence genes as well as serotypes, and whole-genome sequencing was used to analyze the multilocus sequence typing.

**Results:**

The detection rates for the *oqxA* resistance gene and *fimC* virulence gene were highest (93.3%, respectively). There were no correlations between the drug resistance and virulence gene numbers in the same strain. The epidemic serotype was O81 (5/24), ST3856 was an epidemic sequence type, and strains I-9 and III-6 carried 11 virulence genes. The *E. coli* strains from the duck farms in the Zhanjiang area were thus found to have a broad drug resistance spectrum, various virulence genes, complex serotypes, and certain pathogenicity and genetic relationship.

**Discussion:**

Monitoring the spread of pathogenic bacteria and the provision of guidance regarding the use of antibiotics in the livestock and poultry industries will be required in the future in the Zhanjiang area.

## Introduction

*Escherichia coli* is a widespread bacteria that can become pathogenic under certain conditions, which can create a threat to human and animal health. Consequently, in recent years, this has become an important research topic in the field of epidemiology (Srivani et al., 2017). Since the discovery of antibiotics, their long-term overuse has resulted in the gradual development of drug resistance in some bacteria. After drug-resistant bacteria are excreted in livestock and poultry faeces, the drug-resistant genes carried by the bacteria can be transmitted to other bacteria through mobile elements such as plasmids, transposons, and integrons, resulting in their spread and diffusion (Choi et al., 2021). The pathogenicity of *E. coli* is related to its virulence genes, including those encoding adhesins, pathogenicity islands, and outer membrane proteins. When invading the body, numerous virulence genes from *E. coli* interact with each other, and this enables them to escape and destroy the host’s defense mechanism, resulting in host inflammatory response (Ugwu et al., 2020). The O antigen is an important *E. coli* structure, which is located in the side chain of the cell wall lipopolysaccharide. Currently, the O antigen serotype classification is the standard for epidemiological judgment (Liu et al., 2019). The pathogenicity of *E. coli* is not completely consistent with its serotype, and the dominant serotype of *E. coli* is related to regional, temporal, and source differences. *E. coli* can be divided into groups A, B1, B2, and D according to its phylogenetic group classification, where B2 and D are highly pathogenic, B1 is less pathogenic, and A is non-pathogenic (Coura et al., 2015). Multilocus sequence typing (MLST) was first used in the typing of *Neisseria meningitidis* and has been widely used in the molecular typing of other bacteria in recent years. MLST first detects the sequence numbers of 7–10 housekeeping genes, then compares the sequence type (ST) of the bacteria, and uses this ST data to calculate the genetic and evolutionary relationships among strains (Ramadan et al., 2020). Due to scientific and technological progress, MLST is now based on whole genome sequencing (WGS), which greatly improves the accuracy of the method and means that it is becoming increasingly popular in research (Galarce et al., 2021).

Drug-resistant genes have become new environmental pollutants, and they can spread both horizontally and vertically, leading to dramatic increases in the number of drug-resistant bacteria. Antibiotics discharged from the body are scattered in soil, water, or sediment; antibiotic-resistant bacteria and genes are increasing continuously in farms and surrounding environment. In recent years, there have been many reports on drug-resistant genes in *E. coli* of animal origin; however, there are few reports on drug-resistant genes in *E. coli* of environmental origin. In this study, 30 strains of *E. coli* were isolated and identified from soil and sediment samples collected from duck farms in the Zhanjiang area of China. The 30 strains were selected based on their drug-resistant genes, virulence genes, serotypes, phylogenetic group, and MLST analysis to understand the prevalence of *E. coli* and its drug resistance and virulence in this area and to provide a theoretical basis for the prevention and control of colibacillosis.

## Materials and methods

### Sample sources and strains

Samples were collected from three duck farms (farms I, II, and III) in Zhanjiang, China. The soil samples were collected from the surface 1–3 cm soil samples of the pond bank of the farm, and the sediment samples were collected from the surface 1–5 cm of the pond underwater sediment. Five soil samples and 5 sediment samples were collected at each farm. One *E. coli* strain was isolated from each soil and sediment sample, and 30 *E. coli* strains were isolated overall. Standard *E. coli* (ATCC 25922) was provided by a basic veterinary laboratory of Guangdong Ocean University as an internal control for Clinical and Laboratory Standards Institute (CLSI) procedures. The five strains of soil *E. coli* isolated from each duck farm were numbered as 1–5, whereas the five strains of sediment *E. coli* were numbered 6–10. For example, “I-1” represents the first *E. coli* strain isolated from the soil of farm I.

### Isolation, culture, and purification of *E. coli*

The samples were diluted to 10^6^ CFU/ml using 0.1% peptone water, coated on MacConkey medium, and cultured in an incubator at 37 °C for 18h. The distribution and morphological characteristics of the colonies on the MacConkey medium were observed, a single pink typical colony was selected for line purification on eosin Meilan medium, and a single black metal-luster typical colony was selected for secondary purification on eosin Meilan medium. A suspected *E. coli* single colony was then selected and inoculated in a 2-ml tube containing nutritional broth [Beijing Land Bridge Technology Co. LTD, CM106, 2021] and cultured in a 37 °C incubator for 12h; finally, 30% glycerol was added and the sample was stored at −20 °C until further analysis (Sarba et al., 2019).

### Polymerase chain reaction identification of *E. coli*

According to the instructions of the bacterial DNA extraction kit [Tiagen Biochemical Technology (Beijing) Co., Ltd, DP302, 2021], the genomic DNA of each strain was extracted as a template, with reference to the relevant literature (Hu et al., 2011). *E. coli* alkaline phosphatase gene (*phoA*) is the household gene and present in all *E. coli*, which can be used for specificity identification. The preserved strains were identified by polymerase chain reaction (PCR) using the *phoA* gene. Primer information was shown in **Table 1**. The PCR reaction system was 15 μl [DNA template 1 μl, actual master mix [TaKaRa Biomedical Technology (Beijing) Co., Ltd, RR030Q] 13 μl, and upstream and downstream primers (25 μmol/liter) 0.5 μl each]. PCR reaction conditions were as follows: pre-denaturation at 94 °C for 7 min, denaturation at 94 °C for 30 s, annealing at 55 °C for 30 s, and extension at 72 °C for 30 s, and this cycle was repeated 30 times followed by a final extension at 72 °C for 5 min (Niu et al., 2020). After the reaction, a 5 μl amplification product was used for 1.5% agarose gel electrophoresis, and a 622 bp gene sequence length was identified as *E. coli*.

**Table 1:** PCR primer information.

Gene	Primer sequence (5’-3’)	Fragment length/bp	Annealing temperature/ °C	References
*phoA*	F:CGATTCTGGAAATGGCAAAAC R:CGTGATCAGCCCTGACTATGAC	720	60	Hu et al., 2011
*bla_CIT_*	F:TGGCCAGAACTGACAGGCAAA R:TTTCTCCTGAACGTGGCTGGC	462	55	Monstein et al., 2007
*bla_TEM_*	F:TCGCCGCATACACTATTCTCAGAATGA R:ACGCTCACCGGCTCCAGATTTAT	445	50	Monstein et al., 2007
*bla_DHA_*	F:AACTTTCACAGGTGTGCTGGGT R:CCGTACGCATACTGGCTTTGC	405	55	Monstein et al., 2007
*floR*	F:CTGAACACGACGCCCGCTAT R:GGACCGCTCCGCAAACAA	751	60	Cloeckaert et al., 2000
*fexB*	F:ACTGGACAGGCAGGCTTAAT R:CCTGCCCCAAGATACATTGC	319	57	Ying et al., 2019
*OptrA*	F:CTTATGGATGGTGTGGCAGC R:CCATGTGGTTTGTCGGTTCA	310	59	Sun et al., 2021
*aphA1*	F:ATGGGCTCGCGATAATGTC R:CTCACCGAGGCAGTTCCAT	634	60	Saenz et al., 2004
*aac (3*)*-II*	F: GGCGACTTCACCGTTTCT R: GGACCGATCACCCTACGAG	412	54	Alneama et al., 2021
*sul2*	F: CGGCATCGTCAACATAACCT R: TGTGCGGATGAAGTCAGCTC	721	66	Jiang et al., 2019
*tetM*	F: GAGGTCCGTCTGAACTTTGCG R: AGAAAGGATTTGGCGGCACT	915	56	Zhang et al., 2012
*tetC*	F: CTGGGCTGCTTCCTAATGC R: AGCTGTCCCTGATGGTCGT	580	56	Zhang et al., 2012
*ermB*	F:CGAGTGAAAAAGTACTCAACC R:GCCGTGTTTCATTGCTTGATG	557	52	Hardiati et al., 2021
*oqxA*	F:GATCAGTCAGTGGGATAGTTT R:TACTCGGCGTTAACTGATTA	670	51	Hosseini et al., 2016
*qnrS*	F:ACGACATTCGTCAACTGCAA R:TAAATTGGCACCCTGTAGGC	417	53	Wu et al., 2007
*mcr1*	F: CGGTCAGTCCGTTTGTTC R:CTTGGTCGGTCTGTAGGG	309	53	Liu et al., 2016
*mcr2*	F:TGTTGCTTGTGCCGATTGGA R:AGATGGTATTGTTGGTTGCTG	563	65	Xavier et al., 2016
*chuA*	F:GACGAACCAACGGTCAGGAT R:TGCCGCCAGTACCAAAGACA	279	55	Clermont et al., 2000
*yjaA*	F:TGAAGTGTCAGGAGACGCTG R:ATGGAGAATGCGTTCCTCAAC	211	55	Guo et al., 2023
*tspE4.C2*	F:GAGTAATGTCGGGGGCATTCA R:CGCGCCAACAAAGTATTACG	152	55	Clermont et al., 2000

### Detection of drug-resistant phenotype and genes in *E. coli*

Kirby-Bauer method was used to test the antimicrobial susceptibility of *E. coli* isolated from three farms. These experiments were carried out in accordance with guidelines recommended by the Clinical and Laboratory Standards Institute (Clinical and Laboratory Standards Institute (CLSI), 2021). The phenotypic analysis was verified using a standard strain (ATCC 25922). PCR was used to detect related drug-resistant genes. The 16 resistance genes were β-lactam (*bla_CIT_*, *bla_TEM_*), carbapenems (*bla_DHA_*), amidols (*floR*, *fexB*), aminoglycosides (*optrA, aphA1, aac (* Ugwu et al., 2020)*-II*), sulfonamides (*sul2*), tetracycline (*tetM, tetC*), macrocyclic lipids (*ermB*), quinolones (*oqxA, qnrS*), and colistin (*mcr1, mcr2*). The *E. coli* DNA was extracted using a kit [Tiagen Biochemical Technology (Beijing) Co., Ltd, DP302, 2021], and the annealing temperature, reaction system, and reaction conditions (annealing temperature according to primers) for the 16 pairs of drug-resistan genes were referring to the corresponding literature. Primer information was shown in **Table 1**.

### Identification of phylogenetic group

The phylogenetic group genes of the 30 strains of *E. coli* were detected using triple PCR, and the phylogenetic groups were determined based on the existence or deletion of *chuA*, *yjaA*, and *tspE4.C2* genes. Primer information was shown in **Table 1**.

### Study of serotype, virulence gene, and multi-site sequence typing

Virulence factors of *E.coli* include fimil (*papA* and *papC*), adherin (*fimC* and *tsh*), iron uptake system (*iutA* and *iucC*), pathogenicity island (*irp2*, *irpN*, *fyuA*, and *ler*), serum resistance protein (*ompT* and *iss*), capsule polysaccharide (*kpsMII* and *traT*), toxin (*astA*, *vat*, and *cvaC*), hemolysin (*hlyB*), outer membrane protein (*eaeA*), invasitin (*ibeB*), and other related genes.

According to the sample delivery requirements, *E. coli* were cultured overnight in nutritional broth, then washed several times. The whole genome of *E. coli* was sequenced using Illumina NovaSeq PE150 at the Beijing Novogene Bioinformatics Technology Co., Ltd. All good quality paired reads were assembled using the SOAPdenovo (http://soap.genomics.org.cn/soapdenovo.html). The default value for the k-mer was 63, and contigs with > 500 bp were retained. The assembly results were integrated using CISAV20140304 (https://bio.tools/cisa). The serotype, virulence genes, and MLST results were counted using the Centre for Genomic Epidemiology website (http://genomicepidemiology.org/). The ST results were used for cluster analysis using goeBURST of PHYLOVIZ-2.0, and the spliced gene sequences were analyzed using MEGA 7.0 software for making of phylogenetic tree.

## Results

### Bacteria isolation, culture, and identification

The isolated strain showed a single pink colony on MacConkey medium and purplish-black with a metallic luster on the eosin Meilan medium. The 622 bp target bands of the isolated strains were detected by gel electrophoresis of the PCR products, and all 30 isolated strains were confirmed to be *E. coli*.

### Drug resistance detection

The results of resistance of *E. coli* isolates to 23 antibacterial agents were shown in **Table 2**. The resistance rates of Penicillin, Amoxicillin, Ampicillin, Tetracycline, Sulfamethoxa, Chloramphenicol, Flufenicol, and Rifampicin were higher than 70%. The drug-resistant genes in the 30 identified *E. coli* strains were detected using PCR. The dominant drug-resistant genes identified in the samples from farm I included *optrA*, *oqxA*, and *qnrS*; the dominant drug-resistant gene from farm II was *oqxA*; and the dominant drug-resistant gene from farm III was *floR* (**Table 3**). Overall, 93.3% of the strains carried the drug-resistant gene *oqxA*, which was the highest total detection rate, and this was followed by *bla_CIT_*, *bla_TEM_*, *floR*, *optrA*, *sul2*, and *qnrS*, with detection rates of > 60%. *mcr2* was not detected in any of the isolate.

**Table 2:** Resistance of 30 strains of *E.coli* to 23 antimicrobial agents.

Antimicrobial agents	Resistance rate/%			Antimicrobial agents	Resistance rate/%		
	Farm I	Farm II	Farm III		Farm I	Farm II	Farm III
Penicillin	80	100	100	Ciprofloxacin	0	10	0
Amoxicillin	70	90	80	Ofloxacin	0	0	0
Ampicillin	60	100	80	Lomefloxacin	0	40	0
Amtriannan	0	10	10	Sulfamethoxazole	60	90	90
Cefotaxime	20	70	0	Sulfaisoxazole	10	90	90
Ceftriaxone	10	50	0	Chloramphenicolcol	70	90	50
Gentamicin	0	10	0	Flufenicol	80	80	90
Amikacin	0	0	0	Polymyxin B	0	0	0
Streptomycin	0	20	0	Furantoin	00	0	0
Azithromycin	0	0	10	Rifampin	100	100	100
Tetracycline	70	80	10	Fosfomycin	0	0	0
Doxycycline	20	30	50				

**Table 3:** Results of *E. coli* resistance gene detection.

Drug resistance gene	Detected number of farm I/strain	Detected number of farm II/strain	Detected number of farm III/strain	Total detected number/strain	Detected rate/%
*bla_CIT_*	7	8	4	19	63.3
*bla_TEM_*	7	9	7	23	76.7
*bla_DHA_*	4	8	5	17	56.7
*floR*	6	8	9	23	76.7
*fexB*	6	4	4	14	46.7
*optrA*	10	9	6	25	83.3
*aphA1*	5	4	5	14	46.7
*aac(3)-II*	0	2	0	2	0.7
*sul2*	9	8	7	24	80
*tetM*	5	1	1	7	23.3
*tetC*	1	5	1	7	23.3
*ermB*	1	3	1	5	16.7
*oqxA*	10	10	8	28	93.3
*qnrS*	10	7	7	24	80
*mcr1*	6	5	1	12	40
*mcr2*	0	0	0	0	0

### Carrying status of virulence genes

**Figure 1:**
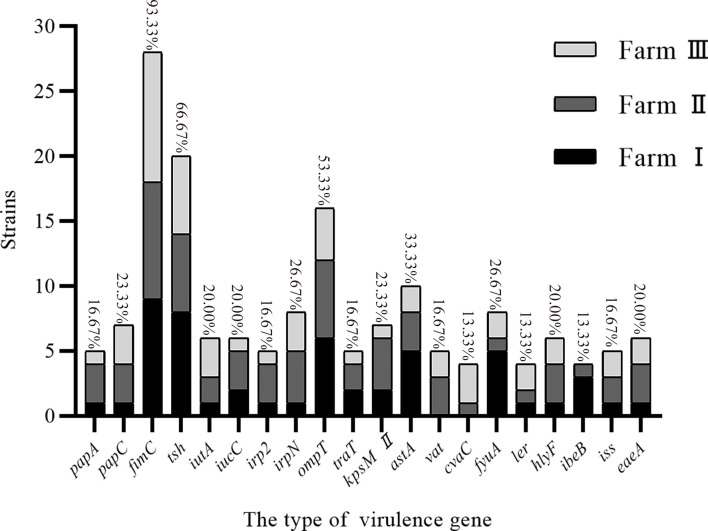
Virulence genes carried by *E. coli*.

**Figure 2:**
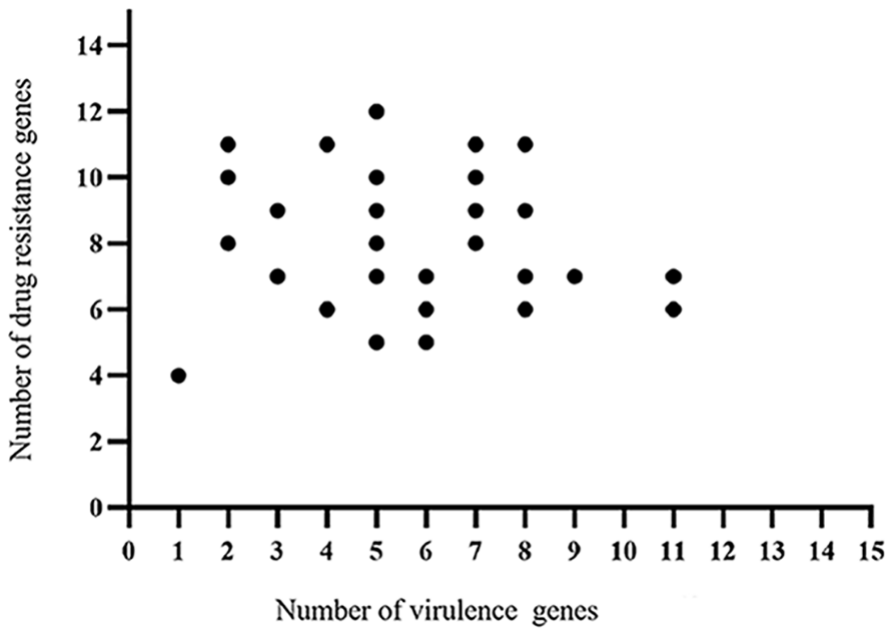
Correlation analysis between the virulence genes and drug-resistant genes identified in the 30 strains *E. coli*.

The whole genome database for the 30 strains of *E. coli* was succesfully sequenced and found to contain 20 different virulence genes (**Figure 1**). Overall, the 30 strains of *E. coli* showed higher carrying rates for three virulence genes, *fimC* (93.3%), *tsh* (66.7%), and *ompT* (53.3%); the carrying rates of *cvaC*, *ler*, and *ibeB* were the lowest (13.3%), and the carrying rates of the remaining 14 virulence genes were 16.7%–33.3%. The scatter plot showed no correlation between the number of drug-resistant genes and virulence genes carried by the same strain (**Figure 2**).

### O antigen serotype of *E. coli*

Serotype analysis showed that 24 strains of *E.coli* were classified, covering 15 serotypes, and no cross-serotypes were detected. There were significant differences in the serotypes among the three duck farms, and only I-9 and III-6 shared a common serotype O9. The most prevalent serotype was O81 (5/24), and this was followed by O174 (2/24), O9 (2/24), O51 (2/24), O98 (2/24), and O86 (2/24) (**Table 4**).

**Table 4:** Serotype results of 30 strains of *E. coli*.

Serotype	Strain	Number of strains / strain
O174	I-2, I-6	2
O35	I-4	1
O176	I-5	1
O39	I-7	1
O142	I-8	1
O9	I-9, III-6	2
O96	I-10	1
O51	II-1, II-6	2
O98	II-3, II-4	2
O18	II-7	1
O86	II-8, II-9	2
O88	II-10	1
O16	III-1	1
O81	III-4, III-5, III-8, III-9, III-10	5
O32	III-7	1

### Phylogenetic group identification

**Figure 3:**
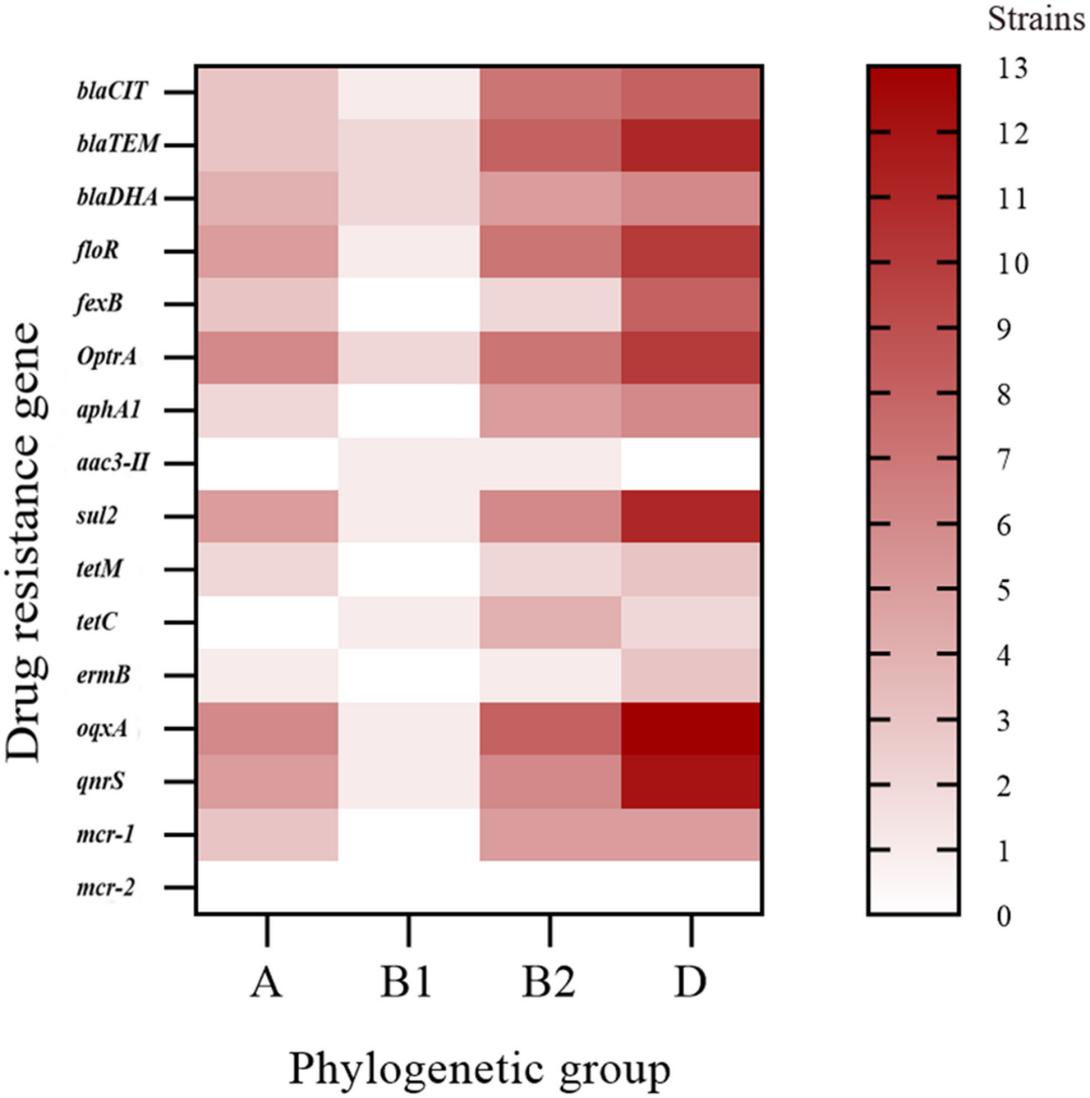
Distribution heat map showing the drug-resistant genes in the different phylogenetic groups.

The phylogenetic groups of the 30 *E. coli* strains were studied using triple PCR. The isolates from farms I and III were mainly from group D, and the isolates from farm II were mainly from group B2 (**Table 5**). Overall, among the 30 isolates, more strains were identified as part of the highly pathogenic groups D (46.7%) and B2 (26.7%), and fewer strains belonged to the non-pathogenic group A (20%) and low pathogenic group B1 (6.7%). Analysis of the correlation between drug-resistant genes and the phylogenetic groups of *E. coli* revealed the types of drug-resistant genes carried by groups B2 and D were more abundant than those carried by groups A and B1 (**Figure 3**). Group B2 isolates mainly carried *bla_TEM_* and *oqxA* but did not carry *mcr2*, whereas group D isolates mainly carried *oqxA*, *qnrS*, *bla_TEM_*, and *sul2* but did not carry *aac* (Ugwu et al., 2020)*-II*, and *mcr2*. *E. coli* carrying the resistance genes *bla_TEM_* and *bla_CIT_* were mainly distributed in groups B2 and D.

**Table 5:** Identification results of phylogenetic groups of *E. coli*.

Group	Strain number of farm I/strain	Strain number of farm II/strain	Strain number of farm III/strain	Total strain number / strain	Total ratio /%
A	3	1	2	6	20
B1	0	1	1	2	6.7
B2	2	5	1	8	26.7
D	5	3	6	14	46.7

### Multilocus sequence typing

Overall, 10 STs were identified from farm I, eight from farm II, and six from farm III (**Table 6**). ST2179 was the most widely distributed ST and was identified on farms I and III, whereas the rest of the ST were only identified at single duck farms. Newly discovered STs (N1–N5) were found on each farm.

**Table 6:** MLST typing, serotypes, virulence genes, resistance and phylogenetic diversity of *E. coli*.

Strain Number	ST type	Serotype	Phylogenetic group	Amplified virulence genes	Phenotype resistance	Amplified resistance genes
I-1	ST1629	–	D	*fimC, ompT, ler, tsh*	PEN, AMX, TET, SIZ, CPL, FFC, RIF, AMP	*bla_CTT,_ bla_TEM,_ floR, fexB, optrA, sul2, tetM, ermB, oqxA, qnrs, mcr1*
I-2	N1	O174	D	*ibeB, fimC, kpsMII, fyuA, tsh*	PEN, CTR, TET, SIZ, CPL, FFC, RIF, AMP	*bla_CTT,_ bla_TEM,_ bla_DHA,_ floR, fexB, optrA, sul2, tetM, tetC, oqxA, qnrs, mcr1*
I-3	ST12233	–	A	*fimC, kpsMII*	PEN, AMX, TET, DOX, SMZ, SIZ, CPL, FFC, RIF, AMP	*bla_CTT,_ bla_DHA,_ floR, fexB, optrA, aphA1, sul2, tetM, oqxA, qnrs, mcr1*
I-4	ST6803	O35	B2	*papA , fimC, ompT, traT, astA, fyuA, tsh*	PEN, AMX, TET, DOX, SMZ, SIZ, CPL, FFC, RIF, AMP	*bla_CTT,_ bla_TEM,_ bla_DHA,_ floR, optrA, aphA1, sul2, tetM, oqxA, qnrs, mcr1*
I-5	ST7013	O176	A	*fimC, ompT*	PEN, AMX, TET, SMZ, SIZ, CPL, FFC, RIF, AMP	*bla_CTT,_ bla_TEM,_ bla_DHA,_ floR, optrA, sul2, tetM, oqxA, qnrs, mcr1*
I-6	N2	O174	B2	*fimC, tsh, ompT, astA, fyuA, ibeB*	AMX, TET, SMZ, SIZ, FFC, RIF	*bla_CTT,_ bla_TEM,_ optrA, oqxA, qnrs, mcr1*
I-7	ST6707	O39	D	*fimC, astA, tsh*	PEN, SIZ, RIF	*bla_TEM,_ floR, optrA, aphA1, sul2, oqxA, qnrs*
I-8	ST3871	O142	D	*fimC, tsh, astA, fyuA, ibeB, irp2, ompT*	CTX, TET, SIZ, CPL, FFC, RIF	*bla_TEM,_ fexB, optrA, aphA1, sul2, oqxA, qnrs*
I-9	ST2179	O9	D	*fimC, iutA, iucC, iroN, traT, astA, fyuA, hlyF, tsh, iss, eaeA*	PEN, AMX, SMZ, SIZ, RIF, AMP	*bla_CTT,_ fexB, optrA, aphA1, sul2, oqxA, qnrs*
I-10	ST10591	O96	A	*papC, fimC, iucC , ompT, tsh*	PEN, AMX, CTX, SMZ, SIZ, CPL, FFC, RIF	*fexB, optrA, sul2, oqxA, qnrs*
II-1	ST6709	O51	D	*fimC, iucC, ompT, kpsMII, astA, iroN, eaeA, tsh*	PEN, AMX, CTX, TET, SMZ, SIZ, CPL, FFC, RIF, AMP	*bla_DHA,_ fexB, optrA, sul2, ermB, oqxA, qnrs*
II-2	ST48	–	B2	*papC, fimC, vat, irpN, ompT*	PEN, AMX, CTX, CTR, TET, CIP, LOM, SMZ, SIZ, FFC, RIF, AMP	*bla_CTT,_ bla_TEM,_ bla_DHA,_ floR, fexB, optrA, aphA1, sul2, tetM, tetC, oqxA, mcr1*
II-3	N3	O98	B2	*papA, fimC, iucC, kpsMII, astA, hlyF, tsh, ompT*	PEN, AMX, CTX, CTR, TET, RIF, AMP	*bla_CTT,_ bla_TEM,_ bla_DHA,_ floR, fexB, optrA, aphA1, sul2, oqxA, qnrs, mcr1*
II-4	ST3107	O98	D	*papC, fimC, irp2, iroN, traT, tsh, eaeA*	PEN, AMX, CTX, STR, TET, DOX, LOM, SMZ, SIZ, CPL, FFC, RIF, AMP	*bla_CTT,_ bla_TEM,_ bla_DHA,_ floR, fexB, optrA, aphA1, sul2, oqxA, qnrs*
II-5	N4	–	B2	*fimC, vat, ibeB , irpN, iucC, irp2, traT, hlyF, tsh*	PEN, AMX, CTX, CTR, STR, TET, LOM, SMZ, SIZ, CPL, FFC, RIF, AMP	*bla_CTT,_ bla_TEM,_ bla_DHA,_ floR, fexB, optrA, aphA1, tetC, ermB, oqxA, qnrs, mcr1*
II-6	ST701	051	B2	*iutA, cvaC, kpsMII, ler, eaeA*	PEN, AMX, CTX, CTR, SMZ, SIZ, CPL, FFC, RIF, AMP	*bla_CTT,_ bla_TEM ,_floR, optrA, aac(3)- II, sul2, tetC, oqxA, qnrs*
II-7	ST226	018	D	*fimC, iutA, irp2, astA, fyuA, hlyF, tsh, iss*	PEN, AMX, SMZ, SIZ, CPL, FFC, RIF, AMP	*bla_CTT,_ bla_TEM,_ bla_DHA,_ floR, optrA, sul2, oqxA, qnrs, mcr1*
II-8	ST6790	086	B2	*papA, fimC, vat, ompT, kpsMII*	PEN, AMX, TET, SMZ, SIZ, CPL, FFC, RIF, AMP	*bla_CTT,_ bla_TEM,_ bla_DHA,_ floR, optrA, sul2, tetC, oqxA*
II-9	ST6790	086	B1	*papA, fimC, ompT, iss*	PEN, AMX, CTX, CTR, ATM, GEN, TET, DOX, LOM, SMZ, SIZ, CPL, RIF, AMP	*bla_TEM,_ bla_DHA,_ optrA, aac(3)- II, tetC, oqxA*
II-10	ST48	O88	A	*papC, fimC, tsh*	PEN, AMX, TET, DOX, SMZ, SIZ, CPL, FFC, RIF, AMP	*bla_CTT,_ bla_TEM,_ bla_DHA,_ floR, optrA, sul2, ermB, oqxA, qnrs, mcr1*
III-1	ST1286	O16	D	*fimC, cvaC, irpN, iutA, iucC, tsh*	PEN, TET, FFC, RIF, AMP	*bla_DHA,_ floR, fexB, oqxA, qnrs, mcr1*
III-2	ST7201	–	D	*fimC, Ler, irpN, ompT, kpsMII, hlyF, tsh*	PEN, AMX, TET, DOX, SMZ, SIZ, FFC, RIF, AMP	*bla_CTT,_ bla_TEM,_ floR, fexB, aphA1, sul2, oqxA*
III-3	ST155	–	D	*papC, fimC, iutA, cvaC, astA*	PEN, AMX, TET, SMZ, SIZ, RIF, AMP	*bla_TEM,_ aphA1, tetM, oqxA, qnrs*
III-4	ST3856	O81	D	*fimC, fyuA, eaeA*	PEN, AMX, TET, SMZ, SIZ, FFC, RIF, AMP	*bla_CTT,_ bla_DHA,_ floR, fexB, aphA1, sul2, tetC, ermB, qnrs*
III-5	ST3856	O81	D	*fimC, tsh, irp2, iss*	PEN, AMX, TET, DOX, SMZ, SIZ, FFC, RIF	*bla_TEM,_ floR, optrA, sul2, oqxA, qnrs*,
III-6	ST2179	O9	D	*papA, fimC, traT, astA, vat, ompT, fyuA, hlyF, tsh, iss, eaeA*	PEN, AMX, TET, DOX, SMZ, SIZ, CPL, FFC, RIF, AMP	*bla_CTT,_ bla_TEM,_ floR, optrA, sul2, oqxA*
III-7	N5	O31	B1	*papC, fimC, vat, ler, tsh*	PEN, AMX, TET, DOX, SMZ, SIZ, CPL, FFC, RIF, AMP	*bla_CTT,_ bla_TEM,_ bla_DHA,_ floR, optrA, sul2, qnrs*
III-8	ST3856	O81	A	*fimC*	PEN, AMX, AZM, TET, SMZ, SIZ, CPL, FFC, RIF, AMP	*bla_TEM,_ floR, optrA, oqxA*
III-9	ST3856	O81	A	*fimC, ompT*	PEN, AMX, ATM, TET, DOX, SMZ, SIZ, CPL, FFC, RIF, AMP	*bla_DHA,_ floR, fexB, optrA, aphA1, sul2, oqxA, qnrs*
III-10	ST3856	O81	B2	*papC, fimC, iutA, ompT, cvaC, tsh, irpN*	PEN, TET, SMZ, SIZ, CPL, FFC, RIF	*bla_TEM,_ bla_DHA,_ floR, optrA ,aphA1, sul2, oqxA, qnrs*

Note: Penicillin (PEN), Amoxicillin (AMX), Aztreonam (ATM), Tetracycline (TET), Doxycycline (DOX), Sulfamethoxazole (SMZ), Sulfafurazole (SIZ), Chloramphenicol (CPL), Florfenicol (FFC), Rifampin (RIF), Ampicillin (AMP), Ciprofloxacin (CIP), Cefotaxime (CTX), Ceftriaxome (CTR), Gentamycin (GEN), Streptomycin (STR), Azithromycin (AZM), Lomefloxacin (LOM).

The 30 *E. coli* strains can be divided into 23 STs, which were ST1629, N1, ST12233, ST6803, ST7013, N2, ST6707, ST3871, ST2179, ST10591, ST6709, ST48, N3, ST3107, N4, ST701, ST226, ST6790, ST1286, ST7201, ST155, ST3856, and N5. Among these, ST3856 (*n* = 5) was the dominant sequence type, accounting for 16.7%, followed by ST2179 (*n* = 2), ST48 (*n* = 2), and ST6790 (*n* = 2), each accounting for 6.7%, while the remaining 19 STs accounted for 3.3% (*n* = 1).

According to the combination of STs, virulence genes and phylogenetic groups, strains I-9 and III-6 both belonged to ST2179, serotype O9, which carried 11 virulence genes, and they were classified into phylogenetic group D. Strains III-4, III-5, III-8, III-9, and III-10 all belonged to ST3856, and their serotype was O81, but their virulence gene types and phylogenetic groups were quite different. Strain III-8 carried only one virulence gene and was the least virulent among the 30 strains of *E. coli*. Different phylogenetic groups were associated with the number of virulence gene types.

### Genetic evolution analysis

**Figure 4:**
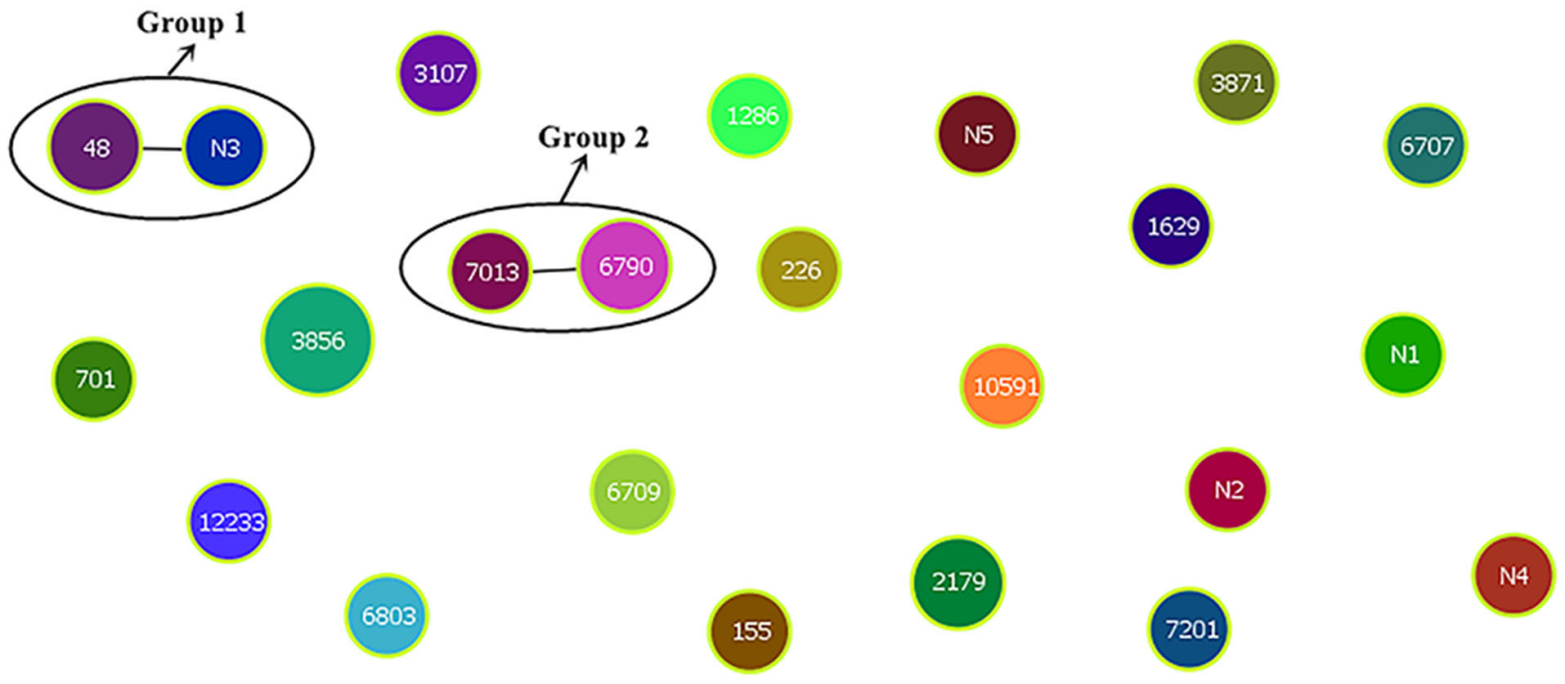
Eburst clustering of 30 *E. coli* genotypes.

Cluster analysis of the ST results was performed using the goeBURST program in PHYLOVIZ-2.0 (**Figure 4**). Each circle represented an ST, the circle size represented the number of strains, and groups 1 and 2 represented two clone groups, respectively. Cluster analysis showed that the 30 isolates were divided into two clonal complexes (CCs) and 19 independent types, the CC1 included ST48 (farm II) and N3 (farm II), and the CC2 included ST7013 (farm I) and ST6790 (farm II).

**Figure 5:**
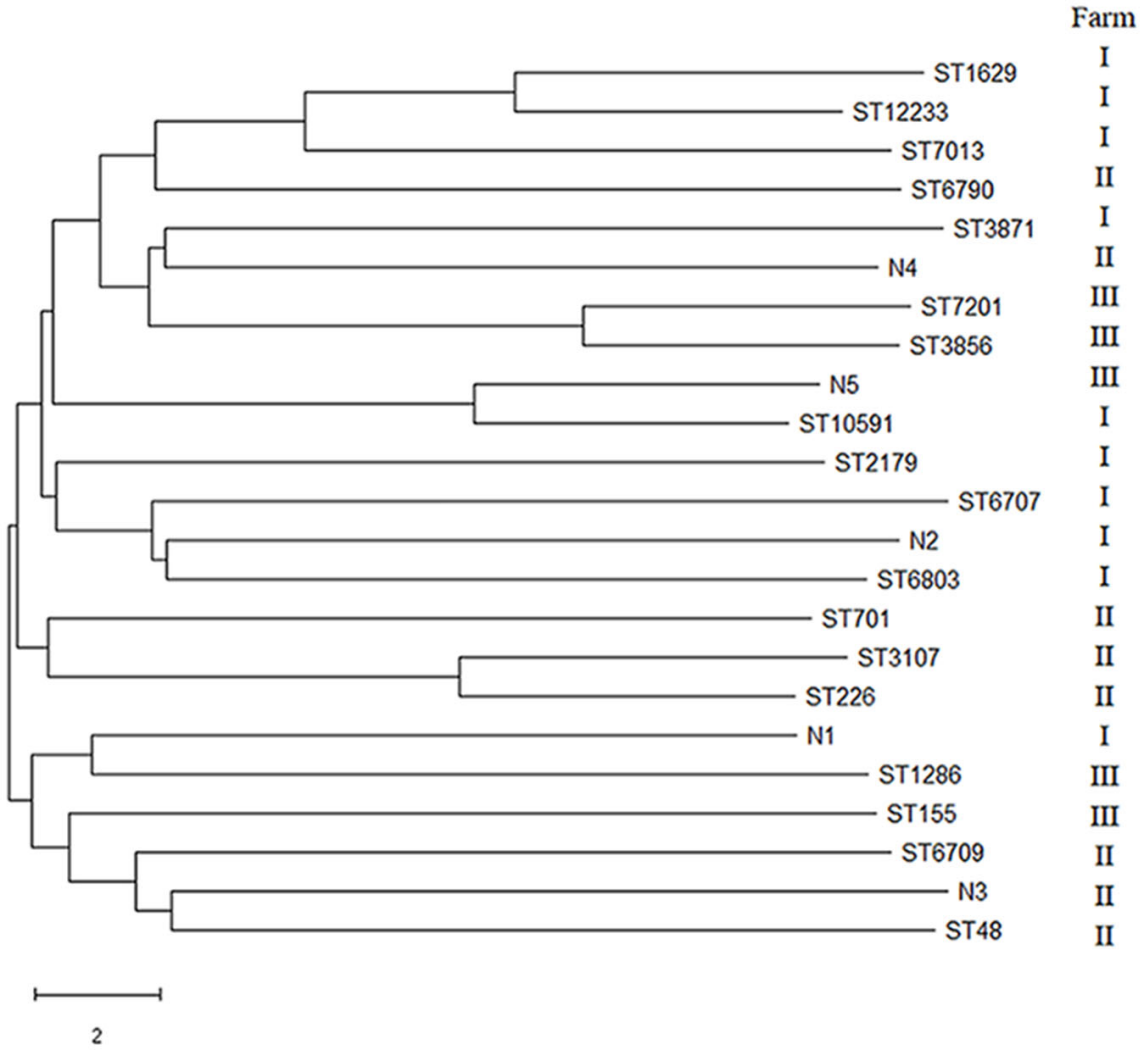
Phylogenetic tree analysis of 30 *E. coli*.

The phylogenetic tree of the STs from the 30 *E. coli* strains was constructed using the neighbor-joining (NJ) method in MEGA7.0. The 30 *E. coli* isolates were divided into six branches: The same duck strains were relatively concentrated in the same branch, and the different duck strains were located in the different branch (**Figure 5**).

## Discussion

### Analysis of the drug resistance results

Drug-resistant genes have become new environmental pollutants, leading to dramatic increases in the number of drug-resistant bacteria. In recent years, there are few reports on drug-resistant genes in *E. coli* of environmental origin. In this study, the resistance rate of *E. coli* isolates to several kinds of antibiotics was higher. The third-generation cephalosporin is a broad-spectrum antibiotic, which has bacteriostatic effect on enterobacterium. The resistance rate of *E. coli* isolated from farm II to Cefotaxime and Ceftriazone was up to more than 50%, which was similar to the study of Guo et al. (2023). This may be related to the frequent use of such drugs in clinic. Excessive or unreasonable use of antibiotics leads to serious drug resistance. Rifampicin is effective against most gram-positive and negative bacteria. *E. coli* isolated from three farms had a 100% resistance rate, which was similar to the results of Zhang; *E. coli* isolated had a 97% resistance rate from duck farms in Sichuan province of China (Zhang et al., 2021). In the study on *Campylobacter* isolated from duck farms in China, the rate of resistance to Chloramphenicol was 42.7% (Han et al., 2019), which was similar to the results of this study, whereas Zendehbad et al. reported that the rate of resistance was 5.3% (Zendehbad et al., 2015), and Jamali reported that the rate of resistance was 4.4% (Jamali et al., 2015). The reason for this phenomenon may be different countries and regions in the treatment of animal diseases or in the addition of antibiotics in feed type and dosage standards.

Thirthy *E. coli* strains were assessed, and 93.3% were found to carry the drug-resistant gene *oqxA*, which was the highest detection rate of all resistance genes assessed. The *oqxA* resistance gene may thus be the dominant drug-resistant gene in duck farms in the Zhanjiang area of China. *oqxA* is an efflux pump that mediates bacterial resistance to quinolones *via* plasmids. It can coexist with other quinolone genes and also spread horizontally among different bacteria (Yamane et al., 2007). Extended spectrum β-lactamases (ESBLs) are the main mechanism of resistance to β -lactamase, and TEM is one of the main genotypes of ESBLs. Mijalli et al (Al-Mijalli, 2016). found that the *bla_TEM_* carrying rate was 20.5% in 75 urine *E. coli* strains isolated from urology clinics in Saudi Arabia, and Lalzampuia et al. (2013) found that the *bla_TEM_* carrying rate was 2.2% in eight ESBL strains from pigs in northeast India. In this study, 76.7% of the *E. coli* isolates carried *bla_TEM_*, which was relatively high, indicating that the epidemic distribution of ESBL-producing strains varied with different regions and sources. In addition, the *SHV*, *CTX-M*, and *OXA* genes have also become a focus of research in relation to ESBL-producing *E. coli*. The present study also explored the presence of other ESBL-producing genes.

### Analysis of virulence genes in *E. coli*

The 30 *E. coli* strains used in this study were found to contain 20 virulence genes, and duck farms I, II, and III carried 18, 20, and 19 virulence genes, respectively. Overall, the 30 *E. coli* strains mainly carried *fimC* (93.3%), *tsh* (66.7%), and *ompT* (53.3%) virulence genes. *fimC* is an adhesion factor virulence gene, *tsh* is an *E. coli* serine protease autotransporter, and *ompT* can cause host diseases by processing or degrading a variety of host proteins (Goudarztalejerdi et al., 2020). This data thus indicated that if the *E. coli* from the duck farms in the Zhanjiang area enters the animal body, it could cause disease, mainly through the action of adhesins, transport systems, and enzymes. Adhesion is the basis of bacterial pathogenicity, and *fimC* is an important component of type I fimbriae. Goudarztalejerdi et al. reported that 87% of *fimC* was detected in 100 avian pathogenic *E. coli* strains and 95% of *fimC* was detected in 100 avian faecal *E. coli* strains in Iran (Goudarztalejerdi et al., 2020). In this investigation, the carrying rate of *fimC* was 93.3%, which is similar to the results reported above. Furthermore, there was no correlation found between the number of drug-resistant genes and virulence genes carried by the same strain.

### Analysis of serotype results

Bacterial serotypes were counted by WGS (Zhang et al., 2015). Among the 30 *E. coli* strains isolated in this study, 24 (80%) were serotyped, and the typing rate was high. There were 15 serotypes with large differences within each duck farm. Only I-9 and III-6 had the same serotype, O9, which proved that the serotypes of strains from different farms in the same area were different, and the same serotype strains probably existed in different farms. O81, O174, O9, O51, O98, and O86 were the epidemic serotypes of the isolates, among which O81 was the most common, and no mixed serotype strains were found. Shimaa et al. found that the dominant serotype of *E. coli* isolated from falcons and Great Spotted Falcons was O158 (Shimaa and Samy, 2016). Thanh et al. isolated 975 strains of *E. coli* from chickens, farm wild animals (ants, geckos, flies, and mice), and the environment and found that the dominant serotype was O18 (Nguyen et al., 2021). The dominant serotypes of the *E. coli* from different sources vary. In addition, some studies have also shown that the *E. coli* serotype is associated with pathogenicity, and thus serotype could help to indicate the pathogenicity of *E. coli*.

### Analysis of phylogenetic group results

Phylogenetic analysis showed that the 30 isolates belonged mainly to groups D (46.7%) and B2 (26.7%). This was different from previous phylogenetic group results for *E. coli* strains, which found that they mainly belonging to group A; such as Rehman et al. who isolated *E. coli* from yaks with diarrhoea in the Qinghai Plateau, China; Rahayuningtyas et al. who isolated 140 strains of *E. coli* from raw sewage in Kuwait; and Nancy et al. who isolated 116 strains of human symbiotic *E. coli*, most of which belonged to group A (Rehman et al., 2017; Stoppe et al., 2017; Rahayuningtyas et al., 2020). A certain association between phylogenetic group and virulence factors has been reproted. Group B2 has the strongest virulence, with more than three virulence genes, followed by group D with two to three virulence genes (Hesam et al., 2015). Our study showed that strains I-9 and III-6 carried the most virulence genes and both belonged to group D, which is different from previous reports. According to the correlation analysis between drug-resistant genes and phylogenetic groups of *E. coli*, the types of drug-resistant genes in groups B2 and D were more abundant than those in groups A and B1, and the numbers of β-lactam resistance genes *bla_TEM_* and *bla_CIT_* in groups B2 and D were greater than those in groups A and B1. It was speculated that the *E. coli* in groups B2 and D could easily acquire drug resistance mechanisms and that there may be a positive correlation between *bla_TEM_* and *bla_CIT_* resistance genes and virulence, which needs to be further explored.

### Analysis of multi-point sequence typing results

Multisite sequence typing reflects the evolutionary biology of bacteria. According to the statistics in our study, the ST of *E. coli* isolated from duck farm III was the least, whereas that from farm I was the most, and all 10 strains had different STs, which indicated that *E. coli* in farm III had good homology and *E. coli* in farm I had poor homology. ST2179 *E. coli* was also detected in farms I and III (I-9 and III-6). Cluster and phylogenetic tree analyses showed that the *E. coli* had certain genetic relationship in different areas and that the *E. coli* had spread across different duck farms. The serotype of the two strains of ST2179 *E. coli* with the most abundant virulence genes was O9. This serotype is common in pig and sheep farms and poses a threat to farmed animals (Chen et al., 2004), indicating that ST2179 *E. coli* is not only widely distributed but also has strong toxicity and pathogenicity. Factors such as time, location, and strain source affect the distribution of the dominant STs. The 30 *E. coli* isolates were divided into 23 STs (including five new STs), with ST3856 being the dominant type. Epidemiological survey data have shown that certain STs are associated with specific diseases. For example, some researchers have found that *E. coli* sequence type ST131 is an epidemic sequence with a high incidence of parenteral pathogenic *E. coli* infection. ST131 *E. coli* can cause urinary tract infections, meningitis, blood infections, and septicemia (Sabiha et al., 2017). Sana et al. found that the dominant ST of 119 ESBLs and carbapenase-producing *E. coli* strains isolated from Islamabad, Pakistan, was ST131 (Ilyas et al., 2021). Seni et al. studied a population of parenterally pathogenic *E. coli* (ExPEC) in Africa and identified different evolutionary branches of ST131 (Seni et al., 2018). However, the relationship between ST131 strain and specific diseases is not absolute, as ST131 was detected in healthy people in Portugal (Rodrigues et al., 2016), and ST131 was not detected in Avian pathogenic *E. coli* (APEC) in Spain (Sola-Gines et al., 2015). At present, the relationship between the *E. coli* ST3856 sequence type and specific diseases has not been determined.

In summary, *E. coli* from aquaculture farms carried abundant drug-resistant genes, and nine kinds of drug-resistant genes were detected to varying degrees, with the highest detection rate of *oqxA* gene, indicating that the *E. coli* from Zhanjiang duck farms was mainly resistant to quinolone antibiotics. Thirty strains of *E. coli* mainly carried *fimC*, *tsh*, and *ompT* virulence genes, indicating that the strains caused the disease mainly through adhesion, transport system or enzyme action. The strains of *E. coli* in farm II carried the most abundant virulence genes, and the virulence effect was diverse. There was no correlation between the number of resistance genes and virulence genes. The serotyping rate of 30 strains of *E. coli* was 80%, and O81 was the dominant serotype. Most of the isolates belonged to groups B2 and D; they carried more drug-resistant gene types than groups A and B1. There were 23 STs in 30 *E. coli* strains, and ST3856 was an epidemic ST. Phylogenetic group and phylogenetic tree showed that there was a certain genetic relationship between strains from different farms. However, this study also has some limitations such as few sampling points and lack of more comprehensive analysis.

## Data availability statement

The original contributions presented in the study are included in the article/supplementary material. Further inquiries can be directed to the corresponding authors.

## Ethics statement

The animal study was reviewed and approved by Ethics Committee of Guangdong Ocean University.

## Author contributions

SL: conceived and designed the experiments. CL: methodology and writing of the original draft. CL and YDa: formal analysis and investigation. CL, ST, and TZ: data curation. JP, YDi, and YM: study conceptualization. JP, YDi, and YM: supervised the study. YDi and YM: writing, review, and editing. All authors contributed to the manuscript and approved the submitted version.
